# Genome comparison of two Coccolithoviruses

**DOI:** 10.1186/1743-422X-3-15

**Published:** 2006-03-22

**Authors:** Michael J Allen, Declan C Schroeder, Andrew Donkin, Katharine J Crawfurd, William H Wilson

**Affiliations:** 1 Plymouth Marine Laboratory, Prospect Place, The Hoe, Plymouth, PL1 3DH, UK; 2 Marine Biological Association, Citadel Hill, Plymouth, PL1 2PB, UK

## Abstract

**Background:**

The *Coccolithoviridae *is a recently discovered family of viruses that infect the marine coccolithophorid *Emiliania huxleyi*. Following on from the sequencing of the type strain EhV-86, we have sequenced a second strain, EhV-163.

**Results:**

We have sequenced approximately 80% of the EhV-163 genome, equating to more than 200 full length CDSs. Conserved and variable CDSs and a gene replacement have been identified in the EhV-86 and EhV-163 genomes.

**Conclusion:**

The sequencing of EhV-163 has provided a wealth of information which will aid the re-annotating of the EhV-86 genome and identified a gene insertion in EhV-163.

## Background

We recently determined the whole genome sequence of the *Coccolithoviridae *strain EhV-86, a giant dsDNA algal virus from the family *Phycodnaviridae *that infects the marine coccolithophorid *Emiliania huxleyi *[1]. Core genes common to nuclear-cytoplasmic large DNA virus (NCLDV) genomes were identified and eight of these genes were used to create a phylogenetic tree in which EhV-86 was placed at the root of the *Phycodnaviridae *[2]. Due to the placement of EhV-86 on a branch distinct from other *Phycodnaviridae *and the presence of six RNA polymerase subunits (unique among the *Phycodnaviridae*) we suggested this genus would eventually be renamed as a subfamily of the *Phycodnaviridae *termed *Coccolithovirinae*.

Strain EhV-86 was originally isolated, along with many others, in 1999 from an *Emiliania huxleyi *bloom in the English Channel [3,4]. In contrast, EhV-163 was isolated from the geographically distinct area of Western Norway during a mesocosm experiment in 2000 [3]. Both virus genomes were initially estimated to be approximately 410 kbp in size. We have subsequently sequenced the entire EhV-86 genome and shown it to be 407, 933 base pairs (bp) [1]. Phylogenetic analysis based on the DNA polymerase gene has previously shown that EhV-163 is distinct from all English Channel strains isolated thus far [3]. In order to gain further insight into both the common and unique relationship these two viruses have with their host, *Emiliania huxleyi*, and their possible placement within a putative subfamily, we have undertaken to sequence a second coccolithovirus genome, EhV-163.

## Results

The sequencing of EhV-86 was hindered by the highly repetitive nature of the genome (three different types of repeat family were identified [5]), which suggested the elucidation, in a much smaller scaled project, of a second closely related strain would be difficult. However, by using a random shotgun approach at first, followed by a second directed approach to fill in missing sequence based on an EhV-86 backbone, we have managed to sequence approximately 322 kbp of the EhV-163 genome in 267 contigs, equating to around 80% of the estimated genome size. This has provided enough genetic information to perform an analysis of the two coccolithovirus genomes. Of the 472 CDSs predicted in the EhV-86 genome [1], from the EhV-163 contigs, full sequence was obtained for 202 CDSs and partial sequence was obtained for a further 182 CDSs. Contigs from EhV-163 were typically between 95–100% identical to EhV-86 sequence (Additional file 1). Regardless of contig size and content (intergenic or genic), EhV-163 contigs aligned with perfect colinearity (except in one case, discussed below) to the EhV-86 genome sequence.

### Highly conserved CDSs

Of the 202 CDSs that had complete sequence, 20 were identical at DNA level and a further 17 were identical at the amino acid level (Additional file 1). These 37 conserved CDSs are distributed throughout the genome; however there are some that appear to be clustered together in 4 regions. CDSs ehv027 (unknown function), ehv028 (putative ligase) and ehv029 (putative membrane protein); ehv135 (putative membrane protein) and ehv136 (unknown function); ehv165 (putative membrane protein), ehv166 (putative RING finger containing protein), ehv167 (RNA polymerase subunit 10) and ehv168 (putative membrane protein); and ehv260 (unknown function), ehv261 (unknown function) and ehv263 (unknown function) are found in these four clusters. The high degree of conservation among these 37 CDSs implies they are under high selection pressure or were recently acquired by the last common ancestor of EhV-86 and EhV-163. Since it has been shown previously that RNA polymerase was present in the ancestral NCLDV prior to the divergence of the *Poxviridae*, *Iridoviridae*, *Asfariviridae*, *Phycodnaviridae *and *Mimiviridae *families, it is likely that for ehv167, at least, the high degree of conservation is due to a high selection pressure [2,5,6]. This also implies that RNA polymerase function is crucial to the infection strategy of coccolithoviruses, providing further evidence for a life style distinct from the other previously sequenced *Phycodnaviridae *(PBCV-1 and ESV-1).

### Gene replacement

**Figure 1:**
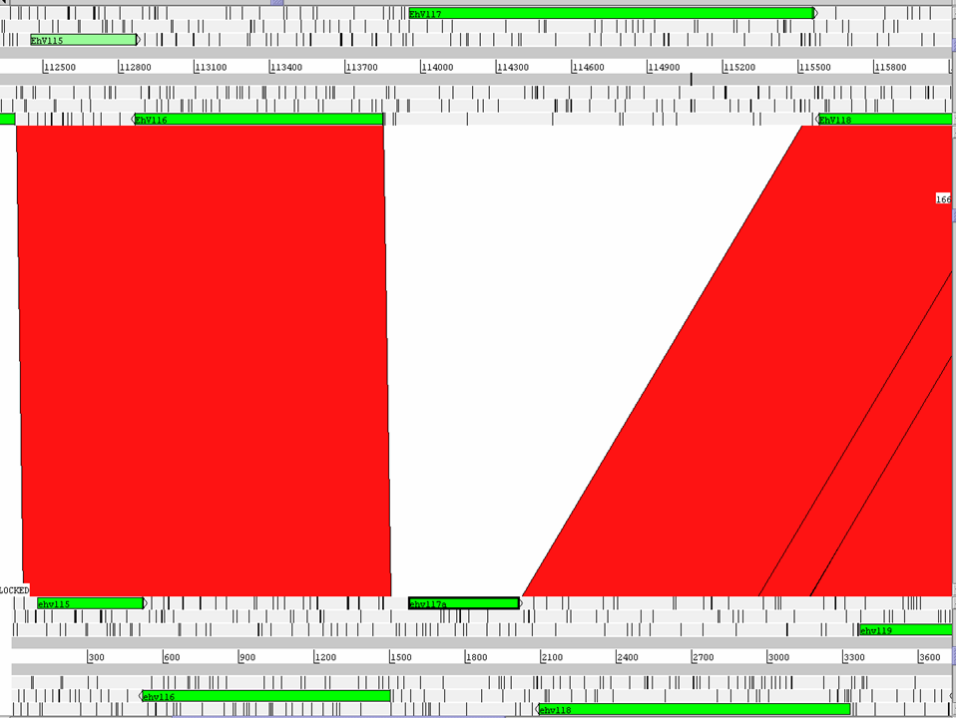
Artemis Comparison Tool (ACT) alignment of EhV-86 genomic sequence (Top) against EhV-163 contig DQ127555 (Bottom). The putative phosphate permease gene, ehv117, of EhV-86 has been replaced by a putative endonuclease, ehv117a, in EhV-163.

No sequence was obtained for 88 of the 472 CDSs predicted to be encoded in the EhV-86 genome. The similar size of the EhV-163 genome in comparison with that of EhV-86 and the high levels of similarity in other regions suggests that the majority of these CDSs are likely to be present. Indeed, a hybridisation of EhV-163 genomic DNA to the EhV-86 based coccolithovirus microarray has revealed that of the 425 EhV-86 CDSs probed for, only 28 appear to be absent in EhV-163 (unpublished data). However, one notable gene deletion in EhV-163 is a putative phosphate permease found at approximately 115 kb on the EhV-86 genome (See Figure 1). This region was sequenced in a 6.9 kbp contig from EhV-163 that contained the full sequence of ehv115, ehv116, ehv118, ehv119, ehv120, ehv121, ehv122 and ehv123. The 1.6 kb phosphate permease gene, known as ehv117, is absent from this contig in EhV-163. This CDS gave no hybridisation signal in the microarray genomic analysis and all attempts to amplify ehv117 by PCR from EhV-163 gDNA have failed (unpublished data). In place of ehv117 in EhV-163 is a 600 bp region that contains a 75 bp 3' remnant of ehv117 and a 435 bp putative CDS that appears to encode a 144 amino acid protein which contains a HNH signature domain, characteristic of a homing endonuclease. The functional relevance of this intriguing gene replacement is yet to be determined and warrants further investigation.

### Variation in CDSs

The majority of EhV-163 CDSs are predicted to start and stop at the same locations as their EhV-86 counterparts. Variation occurs at the DNA and amino acid level but generally the overall length and structure of the genes is very similar. However, there are some differences between the CDSs in the two strains. Changes in DNA sequence can take a variety of forms: point mutations which may or may not lead to the introduction/disruption of the start/stop codon, in-frame insertions/deletions, and insertions/deletions leading to truncated/extended proteins. Examples of all these types of changes can be found when comparing the sequence from the genomes of EhV-86 and EhV-163 (Table 1). The majority of coding inserts and deletions are kept in frame (i.e. occur in multiples of 3 bp). These changes lead to changes in protein structure which could account for different phenotypes (such as host range) to be shown by EhV-163 and EhV-86 [3].

**Table 1:** Examples of genetic changes in the predicted CDSs of EhV-163 in comparison with EhV-86.

**CDS**	**Genetic change(s)**	**Consequence**
ehv060	3' variable region	Truncated protein
ehv100	21 bp deletion	7 amino acid insertion
ehv111	27 bp variable region containing a 3 bp insertion	9 amino acid variable region
ehv118	24 bp and 12 bp insertions	8 and 4 amino acid inserts
ehv128	1 bp insert	Truncated protein
ehv142	Numerous point mutations	Highly variable protein sequence
ehv146	1 bp insertion	Truncated protein
ehv172	12 bp deletion, 1 bp insertion	Truncated protein
ehv173	Two 3 bp deletions, 3 bp and 21 bp insertions	Variable protein sequence
ehv181	24 bp insertion, 15 bp insertion, point mutation creating stop codon	Inserts of 8 and 5 amino acids. Truncated protein.
ehv206	9 bp and 18 bp insertions	Inserts of 3 and 6 amino acids
ehv210A	Point mutation in stop codon	Truncated protein
ehv235	3 bp insertion, 11 bp deletion, numerous small deletions	Truncated protein
ehv276	Point mutation creating stop codon	Truncated protein
ehv277	Point mutation in stop codon	Protein extended
ehv285	1 bp insert	Truncated protein
ehv304	14 bp insert	Truncated protein
ehv321	16 bp insert	Truncated protein
ehv339	Point mutation creating stop codon	Truncated protein
ehv341	Point mutation in stop codon	Protein extended
ehv359	21 bp deletion	7 amino acid deletion
ehv381	Point mutation in start codon, 1 bp deletion	Altered Start of translation
ehv406	Six 1 bp deletions	Variable protein sequence

When annotating a genome it is often necessary to predict where the start of translation codons are. The advantage in having two related genomes is that you can re-check your annotation. This is particularly important in the coccolithoviruses since the majority of CDSs have no database homologues making gene prediction difficult. The vast majority of CDSs in EhV-86 appear to be very similar to their EhV-163 equivalents. However, there are some CDSs that appear to need re-annotating in the light of the sequence data from EhV-163 (Table 1, Additional file 1).

For example, although the overlapping of CDSs is common is some virus genomes [7], this is not a common occurrence in the EhV-86 genome. However, an overlap of CDSs occurs in EhV-86 with ehv380 and ehv381. This overlap does not occur in EhV-163, due to a change in the predicted start of translation methionine codon (ATG to ATA) and a 1 bp deletion that would otherwise cause a frameshift. It therefore appears likely that, in EhV-163 at least, the start of translation occurs from the ATG that is present 36 bp downstream of current predicted ATG start codon of ehv381 in EhV-86.

**Figure 2:**
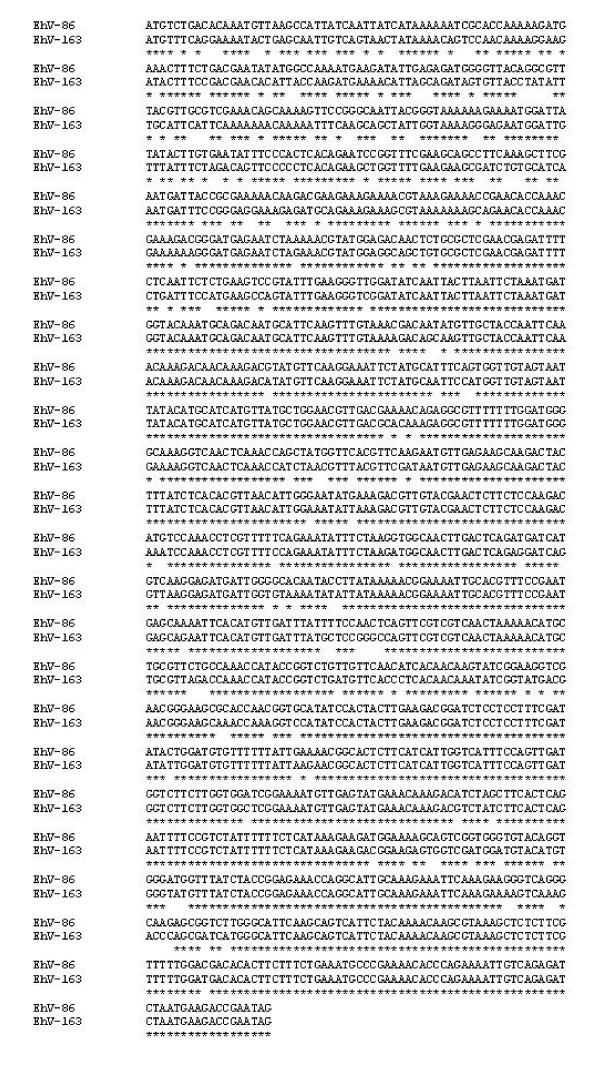
Clustal W alignment of the EhV-86 and EhV-163 homologs for the CDS ehv142. An asterix denotes a conserved base.

There appears to be a high degree of variation in ehv142 between the two strains. The CDS has approximately 86.9 % identity at the nucleotide level (183 of the 1398 nucleotides are different) and 79.1% identity at the amino acid level (97 of the 465 amino acids are different) (Figure 2). Most of the variation occurs in the 5' region of the CDS. BLASTP and PSI-BLAST searches reveal no significant matches. However, PSI-BLAST searches reveal strong matches for KELCH-like proteins (e^-50^) after only two rounds for the EhV-163 version of ehv142. PSI-BLAST searches using the corresponding EhV-86 CDS reveal no matches for KELCH-like proteins, suggesting ehv142 may play a different role in each virus strain. Both EhV-86 and EhV-163 are capable of infecting many of the same strains (with varying virulence) [3]. However, there are many strains of *E. huxleyi *that are susceptible to infection by only one or other of the viruses (unpublished data). Intriguingly, KELCH-like proteins have been identified in poxviruses and are found to be highly variable [8-10]. Indeed, variation in the KELCH-like proteins of poxviruses has been shown to account for variation in virulence, host range and reproduction [8,9].

## Conclusion

EhV-86 and EhV-163 belong to a unique family of algal viruses whose genomes contain a high proportion of genes of unknown function. The sequencing of EhV-163 has provided a wealth of information which will aid the re-annotating of parts of the EhV-86 genome and identified an intriguing gene replacement and a highly divergent CDS in the two genomes. Furthermore, the discovery of highly conserved non-core genes of unknown function in these strains suggests their importance to these viruses, adding further credence to the hypothesis that the Coccolithovirus genus has lifestyle distinct from other members of the *Phycodnaviridae*.

## Methods

### Preparation of EhV-163 concentrate

Six 1L cultures of exponentially growing *E. huxleyi *CCMP1516, at a cell concentration of 1.2 × 10^6 ^cells/ml, were each inoculated with 1 ml of EhV-163 (~2 × 10^5 ^pfu/ml). Growth was monitored by cell counts in a Reichert haemocytometer under a light microscope. Four days post-inoculation, the decimated cultures were subjected to a filtration, concentration and purification regime [3,11].

### Virus DNA extraction

DNA was extracted from CsCl-purified EhV-163 by initially treating the sample with proteinase K (5 mg/ml) in a lysis buffer containing 20 mM EDTA, pH 8.0 and 0.5% SDS (w/v) at 65°C for 1 h. 0.1 × volume aliquots of phenol were added to the samples, after which the DNA was extracted with an equal volume of chloroform:isoamyl alcohol (24:1). The DNA was precipitated with the addition of 0.5 × volume 7.5 M ammonium acetate, pH 7.5 and 2.5 × volume absolute ethanol. Virus DNA was stored in molecular grade water (Sigma) prior to genome sequencing.

### Genome sequencing

Genomic DNA was sheared by sonication, ligated into pCR-Blunt (Invitrogen) and sequenced using M13 forward and reverse primers. After 2700 reads, the sequence was assembled into contigs and analysed using SeqMan (DNAstar). Following alignment to the backbone of EhV-86, 229 primer pairs were designed, specific to the EhV-163 gDNA sequence, to attempt to amplify the missing gaps. The sequence, annealing temperature and genomic location (in relation to EhV-86) of the primers designed can be found in the NERC environmental genomic data catalogue at  under EnvBase accession number egcat:00010. When a PCR product was obtained, it was sequenced directly using both primers and the resulting sequence added to the contig library. The depth of sequence coverage varied across the genome due to the random nature of the initial sequencing strategy. Depth of coverage varied from just one sequence read for some regions to up to18 for others, with an average coverage of approximately 3. In areas of low coverage, sequence reads containing ambiguous results were removed from the analysis. 267 contigs were generated, covering approximately 80% of the EhV-163 genome. These contigs have been submitted to Genbank under the accession numbers DQ127552-DQ127818. This data is also available from , EnvBase accession number egcat:00010.

### Genomic analysis

The Basic Local Alignment Search Tool (BLAST) finds regions of local similarity between sequences by comparing nucleotide or protein sequences to sequence databases and calculating the statistical significance of matches. Protein-protein BLAST (BLAST-P) and Position-specific iterated BLAST (PSI-BLAST) were performed on CDSs of interest online at . Artemis Comparison Tool (ACT) () was used to compare the EhV-163 contigs against the EhV-86 genome.

## Competing interests

The author(s) declare that they have no competing interests.

## Authors' contributions

MJA helped coordinate the study, carried out the molecular genetic studies, sequence alignment and drafted the manuscript. DSCH prepared the EhV-163 DNA for the construction of the shotgun library, helped coordinate the study and draft the manuscript. AD and DSCH constructed the EhV-163 clone library. AD screened the library. AD and KJC performed the sequencing and participated in the sequence alignment. WHW conceived, designed and coordinated the study and helped to draft the manuscript. All authors read and approved the final manuscript.

## Supplementary Material

Additional File 1 Click here for file
