# Supplementary material for: Genome comparison of two Coccolithoviruses
Source: Virol J. 2006 Mar 22;3:15. doi: 10.1186/1743-422X-3-15 (PMC1440845; doi:10.1186/1743-422X-3-15)
Supplement: Additional File 1 [file 1743-422X-3-15-S1.doc]

**Supplementary Table 1.** Comparison of EhV-86 and EhV-163 CDSs for which full sequence was obtained. ^*^When a protein was found to be truncated or extended, similarity was determined using only the overlapping coding region.

| **CDS** | **Function** | **EhV-86**  **bp/aa** | **EhV-163**  **bp/aa** | **Similarity^*^ bp/aa (%)** | **Comment** |
| --- | --- | --- | --- | --- | --- |
| ehv003 | hypothetical protein | 468/155 | 468/155 | 98.5/98.1 |  |
| ehv004 | hypothetical protein | 618/205 | 618/205 | 98.9/98.0 |  |
| ehv008 | hypothetical protein | 489/162 | 489/162 | 95.5/92.6 |  |
| ehv014 | Longevity-assurance (LAG1) family protein | 867/288 | 756/252 | 99.3/94.8 | Later start of translation codon in EhV-163. EhV-86 CDS may need re-annotating. |
| ehv016 | hypothetical protein | 237/78 | 237/78 | 95.4/92.3 |  |
| ehv018 | putative endonuclease | 1077/358 | 1077/358 | 98.6/97.8 |  |
| ehv021 | putative serine protease | 1215/404 | 1101/366 | 96.2/95.6 | Truncated in EhV-163. |
| ehv027 | hypothetical protein | 594/197 | 594/197 | 100/100 | Identical. |
| ehv028 | putative lipase | 792/263 | 792/263 | 100/100 | Identical. |
| ehv029 | putative membrane protein | 633/210 | 633/210 | 100/100 | Identical. |
| ehv030 | DNA polymerase delta catalytic subunit | 3039/1012 | 3039/1012 | 98.6/99.3 |  |
| ehv040 | putative membrane protein | 594/197 | 594/197 | 99.7/99.0 |  |
| ehv043 | hypothetical protein | 1206/401 | 1206/401 | 99.2/98.3 |  |
| ehv044 | hypothetical protein | 288/95 | 288/95 | 100/100 | Identical. |
| ehv045 | putative membrane protein | 465/154 | 408/135 | 97.8/94.8 | Later start of translation codon in EhV-163. EhV-86 CDS may need re-annotating. |
| ehv046 | putative membrane protein | 528/175 | 198/65 | 94.4/89.2 | Truncated in EhV-163. |
| ehv058 | hypothetical protein | 2604/867 | 2604/867 | 99.8/99.8 |  |
| ehv059 | putative membrane protein | 414/137 | 414/137 | 98.1/94.9 |  |
| ehv060 | putative lectin protein | 5985/1994 | 5880/1959 | 98.1/97.2 | Truncated in EhV-163. |
| ehv062 | putative membrane protein | 585/194 | 582/193 | 96.4/95.3 |  |
| ehv063 | hypothetical protein | 390/129 | 390/129 | 99.7/99.2 |  |
| ehv067 | hypothetical protein | 1164/387 | 1188/395 | 93.2/87.6 |  |
| ehv069 | putative membrane protein | 840/279 | 840/279 | 99.4/98.2 |  |
| ehv073 | hypothetical protein | 447/148 | 447/148 | 98.9/98.6 |  |
| ehv074 | putative membrane protein | 564/187 | 564/187 | 99.5/100 | Identical protein. |
| ehv075 | putative membrane protein | 363/120 | 369/122 | 95.9/92.5 |  |
| ehv076 | putative membrane protein | 681/226 | 681/226 | 99.7/100 | Identical protein. |
| ehv077 | putative transmembrane fatty acid elongation protein | 969/322 | 969/322 | 99.3/99.1 |  |
| ehv078 | putative membrane protein | 867/288 | 867/288 | 99.2/98.6 |  |
| ehv079 | putative lipid phosphate phosphatase | 732/243 | 732/243 | 98.5/97.9 |  |
| ehv080 | putative membrane protein | 270/89 | 270/89 | 100/100 | Identical. |
| ehv081 | putative membrane protein | 291/96 | 288/95 | 92.0/80.0 |  |
| ehv082 | putative membrane protein | 528/175 | 528/175 | 99.8/99.4 |  |
| ehv085 | major capsid protein | 1602/533 | 1491/496 | 93.8/99.6 | Later start of translation codon in EhV-163. EhV-86 may need re-annotating. |
| ehv086 | hypothetical protein | 597/198 | 585/194 | 88.4/68.0 | Truncated in EhV-163. Earlier possible start of translation codon in EhV-163. EhV-86 may need re-annotating. |
| ehv092 | hypothetical protein | 546/181 | 546/181 | 99.8/100 | Identical protein. |
| ehv093 | putative HNH endonuclease family protein | 381/126 | 381/126 | 99.0/98.4 |  |
| ehv094 | putative membrane protein | 363/120 | 363/120 | 97.5/95.0 |  |
| ehv095 | putative membrane protein | 1593/530 | 1590/529 |  |  |
| ehv096 | hypothetical protein | 330/109 | 330/109 | 98.8/98.2 |  |
| ehv097 | putative membrane protein | 474/157 | 474/157 | 99.2/99.4 |  |
| ehv098 | hypothetical protein | 348/115 | 348/115 | 97.4/99.1 |  |
| ehv099 | putative membrane protein | 1332/443 | 1332/443 | 95.7/91.2 |  |
| ehv100 | putative membrane protein | 1107/368 | 1074/357 | 90.0/81.5 |  |
| ehv101 | putative hydrolase | 807/268 | 807/268 | 98.6/98.5 |  |
| ehv102 | putative membrane protein | 597/198 | 597/198 | 99.3/98.0 |  |
| ehv103 | putative vesicle associated membrane protein | 354/117 | 354/117 | 98.6/98.3 |  |
| ehv105 | putative transcription factor S (TFIIS) family protein | 495/164 | 453/150 | 99.7/100 | Later start of translation codon in EhV-163. EhV-86 may need re-annotating. |
| ehv106 | hypothetical protein | 1404/467 | 1404/467 | 99.2/99.1 |  |
| ehv110 | putative RING finger protein | 837/278 | 837/278 | 98.7/98.2 |  |
| ehv111 | hypothetical protein | 711/236 | 714/237 | 96.5/94.9 |  |
| ehv114 | putative membrane protein | 342/113 | 216/71 | 98.1/98.6 | Later start of translation codon in EhV-163. EhV-86 may need re-annotating. |
| ehv115 | hypothetical protein | 420/139 | 420/139 | 98.6/98.6 |  |
| ehv116 | putative membrane protein | 984/327 | 984/327 | 98.7/98.2 |  |
| ehv118 | putative membrane protein | 1200/399 | 1236/411 | 95.5/94.7 |  |
| ehv119 | putative membrane protein | 390/129 | 390/129 | 97.2/98.4 |  |
| ehv120 | hypothetical protein | 558/185 | 558/185 | 99.3/98.9 |  |
| ehv121 | hypothetical protein | 762/253 | 762/253 | 98.3/97.6 |  |
| ehv122 | hypothetical protein | 996/331 | 996/331 | 99.2/99.7 |  |
| ehv123 | hypothetical protein | 243/80 | 243/80 | 100/100 | Identical. |
| ehv127 | hypothetical protein | 189/62 | 189/62 | 87.8/80.6 |  |
| ehv128 | thiol oxidoreductase | 510/169 | 495/164 | 97.8/98.8 | Truncated in EhV-163. |
| ehv129 | hypothetical protein | 303/100 | 303/100 | 96.7/96.0 |  |
| ehv130 | hypothetical protein | 420/139 | 426/141 | 97.6/93.5 |  |
| ehv131 | putative membrane protein | 1587/528 | 1581/526 | 94.9/89.9 |  |
| ehv133 | putative ATP dependent protease | 717/238 | 717/238 | 96.8/97.5 |  |
| ehv134 | hypothetical protein | 285/94 | 285/94 | 97.2/93.6 |  |
| ehv135 | putative membrane protein | 318/105 | 318/105 | 100/100 | Identical. |
| ehv136 | putative DNA binding protein | 609/202 | 609/202 | 99.8/100 | Identical protein. |
| ehv139 | hypothetical protein | 435/144 | 435/144 | 99.3/98.6 |  |
| ehv140 | hypothetical protein | 567/188 | 567/188 | 99.3/97.9 |  |
| ehv142 | putative KELCH like protein | 1398/465 | 1398/465 | 86.9/79.1 |  |
| ehv143 | putative membrane protein | 513/170 | 513/170 | 99.0/100 | Identical protein. |
| ehv145 | hypothetical protein | 366/121 | 378/125 | 94.3/92.6 |  |
| ehv146 | putative membrane protein | 756/251 | 648/215 | 98.8/98.6 | Truncated in EhV-163. |
| ehv152 | putative DNA binding protein | 864/287 | 864/287 | 100/100 | Identical. |
| ehv154 | hypothetical protein | 744/247 | 744/247 | 99.2/99.2 |  |
| ehv156 | hypothetical protein | 753/250 | 753/250 | 98.5/98.0 |  |
| ehv157 | putative membrane protein | 282/93 | 282/93 | 99.6/98.9 |  |
| ehv160 | putative serine protease | 1005/334 | 732/243 | 98.4/98.4 | Later start of translation codon in EhV-163. EhV-86 may need re-annotating. |
| ehv165 | putative membrane protein | 879/292 | 879/292 | 99.9/100 | Identical protein. |
| ehv166 | putative RING finger protein | 729/242 | 729/242 | 100/100 | Identical. |
| ehv167 | DNA-directed RNA polymerase subunit | 261/86 | 261/86 | 99.6/100 | Identical protein. |
| ehv168 | putative membrane protein | 510/169 | 510/169 | 99.6/100 | Identical protein. |
| ehv170 | putative membrane protein | 990/329 | 990/329 | 99.5/99.7 |  |
| ehv171 | putative membrane protein | 1278/425 | 1278/425 | 92.8/87.8 |  |
| ehv172 | putative membrane protein | 462/153 | 378/125 | 88.1/81.6 | Truncated in EhV-163. |
| ehv173 | putative membrane protein | 1665/554 | 1683/560 | 86.4/76.4 |  |
| ehv175 | hypothetical protein | 1062/353 | 1062/353 | 99.7/99.7 |  |
| ehv177 | putative membrane protein | 378/125 | 378/125 | 99.7/99.2 |  |
| ehv180 | putative membrane protein | 255/84 | 255/84 | 98.9/100 | Identical protein. |
| ehv181 | putative membrane protein | 684/227 | 612/203 | 90.5/87.2 | Later start of translation codon in EhV-163. EhV-86 may need re-annotating. |
| ehv182 | putative membrane protein | 606/201 | 648/216 | 95.4/93.1 | Contig ends before a stop codon is reached in EhV-163. |
| ehv185 | putative membrane protein | 429/142 | 441/146 | 90.0/85.2 | Truncated in EhV-86. |
| ehv186 | hypothetical protein | 1293/430 | 1293/430 | 99.8/99.8 |  |
| ehv187 | putative membrane protein | 555/184 | 555/184 | 99.1/97.3 |  |
| ehv189 | putative membrane protein | 1200/399 | 1200/399 | 99.2/97.7 |  |
| ehv190 | putative membrane protein | 321/106 | 321/106 | 100/100 | Identical. |
| ehv194 | hypothetical protein | 237/78 | 237/78 | 100/100 | Identical. |
| ehv195 | putative membrane protein | 603/200 | 603/200 | 99.3/98.5 |  |
| ehv198 | hypothetical protein | 345/114 | 345/114 | 100/100 | Identical. |
| ehv202 | hypothetical protein | 231/76 | 231/76 | 99.1/100 | Identical protein. |
| ehv206 | putative membrane protein | 297/98 | 324/107 | 89.9/91.8 |  |
| ehv209 | hypothetical protein | 336/111 | 336/111 | 99.1/99.1 |  |
| ehv210 | hypothetical protein | 735/244 | 735/244 | 99.2/98.4 |  |
| ehv210a | hypothetical protein | 324/107 | 105/34 | 97.1/94.1 | Truncated in EhV-163. |
| ehv211 | hypothetical protein | 390/129 | 390/129 | 96.7/95.3 |  |
| ehv213 | putative membrane protein | 309/102 | 309/102 | 98.7/98.0 |  |
| ehv214 | putative membrane protein | 558/185 | 585/194 | 92.1/87.6 |  |
| ehv215 | hypothetical protein | 480/159 | 480/159 | 99.6/100 | Identical protein. |
| ehv217 | hypothetical protein | 1188/395 | 1185/394 | 96.3/93.1 |  |
| ehv218 | hypothetical protein | 945/314 | 945/314 | 98.6/98.7 |  |
| ehv220 | hypothetical protein | 879/292 | 879/292 | 97.8/97.9 |  |
| ehv230 | putative endonuclease | 387/128 | 387/128 | 96.9/97.7 |  |
| ehv232 | putative membrane protein | 549/182 | 549/182 | 99.1/98.9 |  |
| ehv233 | hypothetical protein | 594/197 | 594/197 | 97.5/97.5 |  |
| ehv234 | hypothetical protein | 1152/383 | 1149/382 | 99.2/99.0 |  |
| ehv235 | hypothetical protein | 633/210 | 435/144 | 90.6/88.9 |  |
| ehv237 | hypothetical protein | 555/184 | 555/184 | 98.9/97.8 |  |
| ehv239 | hypothetical protein | 225/74 | 225/74 | 98.7/97.3 |  |
| ehv251 | hypothetical protein | 660/219 | 660/219 | 99.7/100 | Identical protein. |
| ehv252 | hypothetical protein | 372/123 | 267/88 | 99.6/98.9 | Later start of translation codon in EhV-163. EhV-86 may need re-annotating. |
| ehv254 | hypothetical protein | 351/116 | 351/116 | 98.0/96.6 |  |
| ehv256 | hypothetical protein | 1188/395 | 1188/395 | 99.0/99.0 |  |
| ehv257 | hypothetical protein | 102/33 | 102/33 | 99.0/97.0 |  |
| ehv258 | hypothetical protein | 216/71 | 216/71 | 98.6/97.2 |  |
| ehv260 | hypothetical protein | 453/150 | 453/150 | 99.8/100 | Identical protein. |
| ehv261 | hypothetical protein | 162/53 | 162/53 | 100/100 | Identical. |
| ehv262 | hypothetical protein | 273/90 | 273/90 | 99.6/98.9 |  |
| ehv263 | hypothetical protein | 375/124 | 375/124 | 99.5/100 | Identical protein. |
| ehv264 | hypothetical protein | 543/180 | 543/180 | 98.7/98.9 |  |
| ehv265 | hypothetical protein | 930/309 | 906/301 | 96.9/96.7 |  |
| ehv267 | hypothetical protein | 450/149 | 450/149 | 98.4/95.3 |  |
| ehv268 | putative membrane protein | 720/239 | 720/239 | 94.6/87.0 |  |
| ehv269A | putative membrane protein | 264/87 | 264/87 | 95.5/85.1 |  |
| ehv270 | putative membrane protein | 303/100 | 297/98 | 91.6/84.7 |  |
| ehv272A | hypothetical protein | 321/106 | 339/113 | 97.8/88.7 | Truncated in EhV-86. Contig ends before a stop codon is reached in EhV-163. |
| ehv273 | hypothetical protein | 264/87 | 264/87 | 98.1/95.4 |  |
| ehv274 | hypothetical protein | 489/162 | 489/162 | 97.8/96.3 |  |
| ehv276 | hypothetical protein | 240/79 | 180/59 | 98.9/100 | Truncated in EhV-163. |
| ehv277 | putative membrane protein | 411/136 | 513/170 | 89.8/82.4 | Extended in EhV-163. |
| ehv279 | hypothetical protein | 513/170 | 513/170 | 96.3/94.7 |  |
| ehv280 | hypothetical protein | 528/175 | 528/175 | 98.3/97.7 |  |
| ehv281 | hypothetical protein | 423/140 | 423/140 | 96.7/95.0 |  |
| ehv283 | hypothetical protein | 219/72 | 219/72 | 97.7/97.2 |  |
| ehv284 | hypothetical protein | 315/104 | 315/104 | 97.1/95.2 |  |
| ehv285 | hypothetical protein | 297/98 | 84/27 | 98.8/81.5 | Truncated in EhV-163. |
| ehv288 | hypothetical protein | 540/179 | 543/180 | 93.9/96.1 | Later start of translation codon in EhV-163. EhV-86 may need re-annotating. |
| ehv289 | hypothetical protein | 591/196 | 600/199 | 93.7/91.8 |  |
| ehv293 | hypothetical protein | 666/221 | 447/148 | 96.6/96.6 |  |
| ehv293A | hypothetical protein | 600/199 | 588/196 | 88.5/68.3 | Contig ends before a stop codon is reached in EhV-163. |
| ehv297 | hypothetical protein | 390/129 | 390/129 | 96.9/95.3 |  |
| ehv299 | hypothetical protein | 306/101 | 306/101 | 98.0/100 | Identical protein. |
| ehv300 | hypothetical protein | 426/141 | 426/141 | 96.9/97.2 |  |
| ehv302 | hypothetical protein | 555/184 | 552/183 | 95.5/90.7 |  |
| ehv303 | putative membrane protein | 597/198 | 597/198 | 100/100 | Identical. |
| ehv304 | hypothetical protein | 306/101 | 309/102 | 93.9/82.4 |  |
| ehv305 | hypothetical protein | 489/162 | 489/162 | 97.1/96.3 |  |
| ehv306 | hypothetical protein | 294/97 | 294/97 | 94.6/95.9 |  |
| ehv313 | hypothetical protein | 459/152 | 234/77 | 98.3/98.7 | Extended in EhV-86. |
| ehv314 | hypothetical protein | 516/171 | 516/171 | 100/100 | Identical. |
| ehv318 | putative membrane protein | 177/58 | 63/20 | 96.8/70.0 | Truncated in EhV-163. |
| ehv321 | putative membrane protein | 411/136 | 42/13 |  | Truncated in EhV-163. |
| ehv325 | putative membrane protein | 429/142 | 429/142 | 98.4/97.9 |  |
| ehv331 | hypothetical protein | 462/153 | 462/153 | 100/100 | Identical. |
| ehv336 | hypothetical protein | 282/93 | 282/93 | 98.2/98.9 |  |
| ehv337 | hypothetical protein | 480/159 | 480/159 | 97.5/96.9 |  |
| ehv338 | hypothetical protein | 594/197 | 594/197 | 98.5/96.4 |  |
| ehv339 | hypothetical protein | 879/292 | 204/67 | 94.1/88.1 | Truncated in EhV-163. ehv339A may need annotating. |
| ehv339A | hypothetical protein | 489/162 | 480/159 | 94.0/90.6 | 3’ portion of EhV-86 ehv339. |
| ehv340 | putative membrane protein | 399/132 | 399/132 | 98.2/98.5 |  |
| ehv341 | putative membrane protein | 492/163 | 645/214 | 96.5/93.9 | Truncated in EhV-86. |
| ehv347 | putative membrane protein | 447/148 | 441/146 | 92.5/84.2 |  |
| ehv351 | hypothetical protein | 585/194 | 585/194 | 98.6/98.5 |  |
| ehv352 | hypothetical protein | 357/118 | 357/118 | 95.5/91.5 |  |
| ehv353 | hypothetical protein | 474/157 | 474/157 | 98.5/99.4 |  |
| ehv354 | hypothetical protein | 1839/612 | 1839/612 | 98.3/98.2 |  |
| ehv355 | hypothetical protein | 774/257 | 774/257 | 98.4/97.7 |  |
| ehv358 | thioredoxin | 477/158 | 477/158 | 96.4/96.8 |  |
| ehv359 | putative membrane protein | 522/173 | 501/166 | 95.0/94.0 |  |
| ehv360 | hypothetical protein | 648/215 | 648/215 | 95.8/95.8 |  |
| ehv362 | putative membrane protein | 258/85 | 258/85 | 100/100 | Identical. |
| ehv365 | hypothetical protein | 348/115 | 348/115 | 99.4/99.1 |  |
| ehv367 | hypothetical protein | 465/154 | 465/154 | 98.1/97.4 |  |
| ehv368 | hypothetical protein | 942/313 | 942/313 | 99.6/99.0 |  |
| ehv369 | hypothetical protein | 423/140 | 423/140 | 100/100 | Identical. |
| ehv374 | putative membrane protein | 318/105 | 318/105 | 95.6/90.5 |  |
| ehv375 | putative membrane protein | 570/189 | 570/189 | 99.8/100 | Identical protein. |
| ehv379 | putative membrane protein | 342/113 | 342/113 | 98.8/98.2 |  |
| ehv380 | putative membrane protein | 378/125 | 384/127 | 97.6/95.2 |  |
| ehv381 | putative membrane protein | 258/85 | 222/73 | 99.1/98.6 | Later start of translation codon in EhV-163. EhV-86 may need re-annotating. |
| ehv382 | putative membrane protein | 357/118 | 357/118 | 98.6/97.5 |  |
| ehv383 | putative membrane protein | 390/129 | 390/129 | 97.2/95.3 |  |
| ehv389 | hypothetical protein | 519/172 | 519/172 | 99.6/100 | Identical protein. |
| ehv406 | hypothetical protein | 513/170 | 507/168 | 96.7/85.3 |  |
| ehv407 | putative membrane protein | 486/161 | 486/161 | 98.1/98.8 |  |
| ehv425 | putative membrane protein | 318/105 | 318/105 | 99.1/98.1 |  |
| ehv433 | putative membrane protein | 342/113 | 342/113 | 98.2/98.2 |  |
| ehv445 | hypothetical protein | 486/161 | 486/161 | 100/100 | Identical. |
| ehv446 | hypothetical protein | 414/137 | 414/137 | 99.3/97.8 |  |
| ehv447 | putative serine protease | 906/301 | 906/301 | 100/100 | Identical. |
| ehv449 | hypothetical protein | 489/162 | 489/162 | 99.8/99.4 |  |
